# A 5.86 Million Quality Factor Cylindrical Resonator with Improved Structural Design Based on Thermoelastic Dissipation Analysis

**DOI:** 10.3390/s20216003

**Published:** 2020-10-22

**Authors:** Libin Zeng, Yiming Luo, Yao Pan, Yonglei Jia, Jianping Liu, Zhongqi Tan, Kaiyong Yang, Hui Luo

**Affiliations:** College of Advanced Interdisciplinary Studies, National University of Defense Technology, Changsha 410073, China; zenglibin19@nudt.edu.cn (L.Z.); luoyiming11@nudt.edu.cn (Y.L.); panyao08@nudt.edu.cn (Y.P.); jiayonglei17@nudt.edu.cn (Y.J.); l_jianp@sina.com (J.L.); zqtan@nudt.edu.cn (Z.T.); luohui.luo@163.com (H.L.)

**Keywords:** cylindrical resonator, quality factor, thermoelastic dissipation

## Abstract

The cylindrical resonator is the core component of cylindrical resonator gyroscopes (CRGs). The quality factor (Q factor) of the resonator is one crucial parameter that determines the performance of the gyroscope. In this paper, the finite element method is used to theoretically investigate the influence of the thermoelastic dissipation (TED) of the cylindrical resonator. The improved structure of a fused silica cylindrical resonator is then demonstrated. Compared with the traditional structure, the thermoelastic Q (Q_TED_) of the resonator is increased by 122%. In addition, the Q factor of the improved cylindrical resonator is measured, and results illustrate that, after annealing and chemical etching, the Q factor of the resonator is significantly higher than that of the cylindrical resonators reported previously. The Q factor of the cylindrical resonator in this paper reaches 5.86 million, which is the highest value for a cylindrical resonator to date.

## 1. Introduction

A Coriolis vibratory gyroscope (CVG) measures angular velocity or the angle of a moving object through the precession of the elastic wave of a resonator vibrating in eigenmodes. With a great number of advantages, including high accuracy, considerable stability, long durability, low power consumption and maintenance-free features, CVGs with axisymmetric shell resonators are widely used in navigation fields and platform stabilization systems [1,2,3,4,5,6,7,8]. It is claimed that the well-known Northrop Grumman hemispherical resonator gyroscope (HRG) has continuously worked for over 50 million hours without a single failure [9]. However, its complex structure and high cost have limited the application of these HRGs to high-value space programs. By comparison, the innovative structure of the flat electrode carrier proposed by Sagem has led to the simplification and lowered cost of HRG manufacturing, and, therefore, the massive application of HRGs [10,11,12]. The microfabricated HRGs reported by the University of Michigan and the University of California, Irvine, have achieved similar performance at a much lower cost [13,14,15]. The successful designs and applications have stimulated renewed interest in investigating this type of angular sensor, and HRG model and design [16,17], control strategies [18,19,20], error analysis [21], etc., have since been extensively studied.

Cylindrical resonator gyroscopes (CRGs) are a type of vibratory gyroscope with a symmetrical cylindrical shell resonator. Similar to HRGs, the cylindrical resonator is the core element of the CRG, and its quality factor (Q factor) and symmetry set the limit of the CRG sensitivity and noise performance. Compared with hemispherical resonators, cylindrical resonators are easier to manufacture and can maintain relatively high Q factors. In the manufacturing process, hemispherical resonators require high concentricity for thin spherical shells, which demand high-precision multiple-axis machining centers, complicated manufacturing processes of rough grinding and fine grinding, and relatively long manufacturing times. By contrast, cylindrical resonators only require concentricity for the cylindrical shells and only need a grinding machine, relatively less manufacturing steps [22], and less machining time. Our research group fabricated fused silica cylindrical resonators with the Q factor approaching 8 × 10^5^ in 2016 [23] and 2.89 × 10^6^ in 2019 [24]. Improving the Q factor of the resonator is critical to further improving the overall performance of the CRG.

Q factor is known to be affected by many different kinds of dissipation, including air damping, surface loss, anchor loss and thermoelastic dissipation, etc. Air damping is a loss mechanism caused by friction when a resonator operates in a viscous fluid. When the resonator vibrates, some momentum of the resonator is transferred into the fluid by collision [25]. The air damping can be significantly reduced by operating the resonator in a vacuum environment. Surface loss is mainly caused by defects and impurities on the surface of the resonator and has a great influence on the Q factor of micro-sized resonators, such as MEMS gyroscopes [26]. The mechanism of surface loss has not been explained well in the present research. However, several experiments have actually illustrated that annealing and chemical etching of the resonator can effectively reduce the surface loss [27]. Anchor loss is caused by the mechanical waves generated when the resonator vibrates, which propagate through the anchor stem to the substrate [28]. In order to prevent and reduce the propagation of elastic waves to the substrate, the resonator should be correctly designed to ensure that the anchor stem of the resonator is at a node of the mode on the resonator.

Thermal elastic damping (TED) is derived from the coupling between the mechanical and thermal domains. During vibration, different regions of the resonator experience compressive stress and tensile stress, respectively. Compressive and tensile stresses produce temperature gradients in the resonator. To restore thermal equilibrium, the heat in hot regions flows irreversibly to cold regions, and this process is known as TED [29]. TED is determined by the material, geometry and operating temperature of the resonator. Numerous studies of TED have been reported so far. Zener proposed a general calculation model for the thermoelastic Q (Q_TED_) of wires and reeds, which considered one-dimensional heat transfer in the direction of bending [30]. Braginsky et al. analyzed the effect of surface defects of quartz resonators on TED, which was based on Zener’s model [31]. Shiari et al. simulated the influence of surface roughness of the beam micro-resonators [32], and Sorenson et al. analyzed the effect of surface roughness of micromachined hemispherical shell resonators [33]. Their studies showed that the TED increased with the increase of resonator roughness. In addition, they illustrated that the surface roughness of the resonator had a more significant effect on the TED with larger thickness. Darvishian also comprehensively analyzed and simulated the influence of geometry, materials, rim condition and operating temperature on the TED of micromachined hemispherical shell resonators [34]. Hao and Ayazi established an analytical model for the TED of ring structures and studied the effect of ring dimension on the Q factor due to TED [35]. Hamza used the influence of TED to reduce the Q factor mismatch of the MEMS disk resonator [36]. However, studies on the TED of different cylindrical resonator structures are rather rare, and this study intends to fill this gap.

In this paper, the Q_TED_ and temperature gradient distribution of the cylindrical resonator are first simulated. The direction of irreversible heat flow transfer in the cylindrical resonator is analyzed through the temperature gradient distribution. Based on the simulation results, the structure of the cylindrical resonator is improved to increase the Q_TED_. Then, the Q factor and resonant frequency of the fabricated cylindrical resonator with the improved structure after annealing and chemical etching are reported, respectively, and the Q factor of the cylindrical resonator reaches 5.86 million. To the authors’ best knowledge, this is the highest Q factor value reported for fused silica cylindrical resonators to date.

## 2. Simulation Model

In the process of vibration, it is assumed that the length of the neutral layer of the cylindrical resonator does not change. However, materials on each side of the neutral layer can form a temperature gradient because of the compressive and tensile stresses. The temperature gradient leads to energy transformation from the high-temperature region to the low-temperature region, causing irreversible energy loss. The three-dimensional equation of elastic motion can be written as [30]:

(1) $$\frac{E}{2\left(1+\nu \right)}\nabla^{2}u+\frac{E}{2\left(1+\nu \right)\left(1-2\nu \right)}\nabla \left(\nabla \cdot u\right)-\frac{E\alpha }{2\left(1-2\nu \right)}\nabla T+f=\rho \frac{\partial^{2}u}{\partial t^{2}}$$

where *E* is Young’s modulus in MPa, ν is Poisson’s ratio, α is the coefficient of thermal expansion in 1/K, *f* is the external force per volume in N/m^3^, *T* is the temperature in K and u = (*u, v, w*) represents the deformation vector, respectively. The thermal dynamics can be written as [30]:

(2) $$k\nabla^{2}T-\rho C_{sp}\frac{\partial T}{\partial t}=\frac{E\alpha T}{1-2\nu }\frac{\partial }{\partial t}\nabla \cdot u$$

where *k* represents the thermal conductivity in W/(m K) and *C_sp_* represents the specific heat capacity of the material in J/(kg K). In order to solve these two equations, a finite element-based approach can be applied for arbitrary 3D geometries and finding deformations.

**Figure 1:**
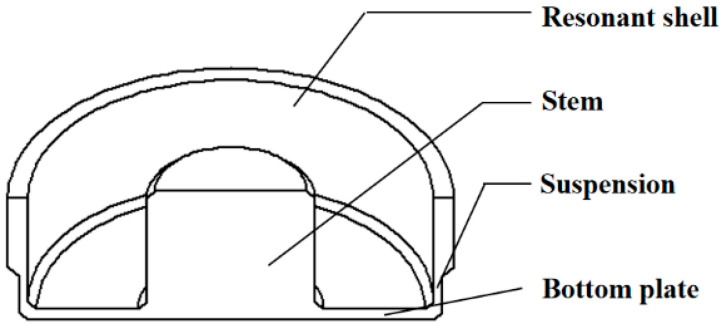
The schematic of the cylindrical resonator structure.

**Figure 2:**
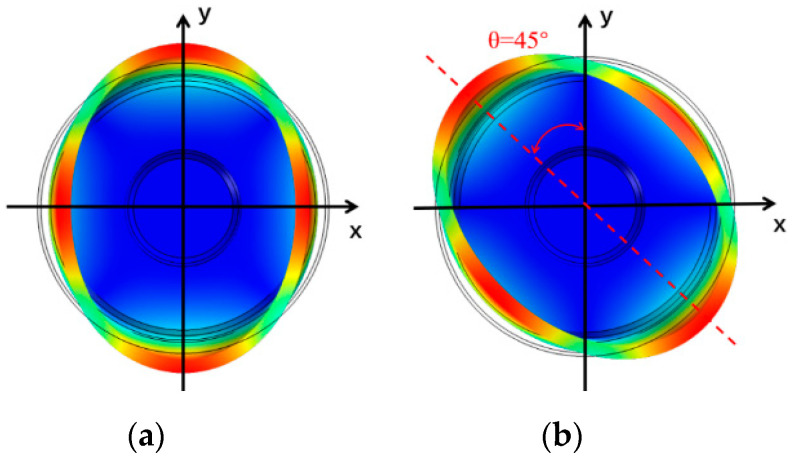
The *n* = 2 mode of the cylindrical resonator: (**a**) Primary mode; (**b**) Secondary mode.

The structure of the cylindrical resonator is shown in Figure 1. COMSOL software is used to analyze the influence of different structures on the TED of cylindrical resonators. A cylindrical resonator operating in the *n* = 2 mode (wineglass mode) is used for angular measurement. Ideally, this mode consists of two degenerate modes that have the same natural frequency and are spatially orthogonal. Each degenerate mode has 4 antinodes and 4 nodes [4], as shown in Figure 2. In the simulation, we assume that TED is the main loss mechanism of the cylindrical resonator; thus, the *Q_TED_* can be calculated by Equation (3) [37]:

(3) $$Q_{TED}=\frac{1}{2}\left|\frac{Re\left(\omega \right)}{Im\left(\omega \right)}\right|$$

where *Q_TED_* represents the Q factor of the TED and ω represents the resonant frequency of *n* = 2 mode.

### 2.1. Simulation

The material of the cylindrical resonator is defined as fused silica. The physical properties of fused silica used for the following simulation are presented in Table 1.

**Figure 3:**
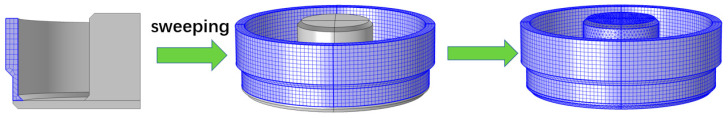
Mesh configuration of the cylindrical resonator.

In the initial structure, the thickness of the resonant shell, suspension and bottom plate was 0.8, 0.3 and 0.6 mm, respectively. In order to minimize the calculation cost and the frequency difference between the two degenerate modes caused by the inhomogeneity of the mesh process, the method of free quadrilateral meshing and sweep meshing was utilized for the cylindrical shell. The rest of the parts were meshed with free tetrahedrons, as shown in Figure 3.

**Figure 4:**
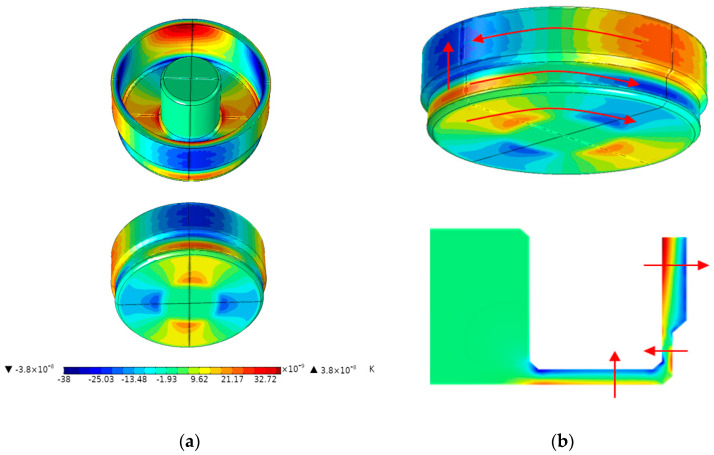
Schematic of: (**a**) The temperature gradient distribution of the cylindrical resonator vibrating at the temperature of T = 293.15 K; (**b**) The directions of the irreversible heat flow.

During the simulation, the cylindrical resonator is operated at the temperature of T = 293.15 K. The end-face of the stem is fixed, and the rest part is in a free vibration state. The temperature gradient distribution of the cylindrical resonator vibrating at this temperature can be obtained by simulation, as shown in Figure 4a. The eigenfrequency is 6039.3 Hz, and the Q_TED_ is 8.14 × 10^8^. A large area of the temperature gradient is formed on the resonant shell, the suspension and the bottom plate, and the resulting heat transfer in these hot and cold regions causes a large amount of TED. In our simulation, there are mainly seven directions of irreversible heat flow: the thickness direction and the circumferential direction of the resonant shell, the suspension and the bottom plate and the longitudinal direction between the resonant shell and the suspension, as shown in Figure 4b.

### 2.2. Effect of Shell Structure

The main way to reduce TED is to reduce the irreversible heat flow, that is, to increase the thermal resistance of the resonator. Thermal resistance indicates the ability of the structure to resist the heat flow, which can be calculated by Equation (4):

(4) $$R=\frac{L}{kS}$$

where *R* represents the thermal resistance, *L* represents the structure thickness in the direction of the heat flow, *k* represents the thermal conductivity and *S* represents the area of heat conduction. Therefore, increasing the thickness of the structure in the direction of heat flow can increase the thermal resistance, that is, reduce the TED.

**Figure 5:**
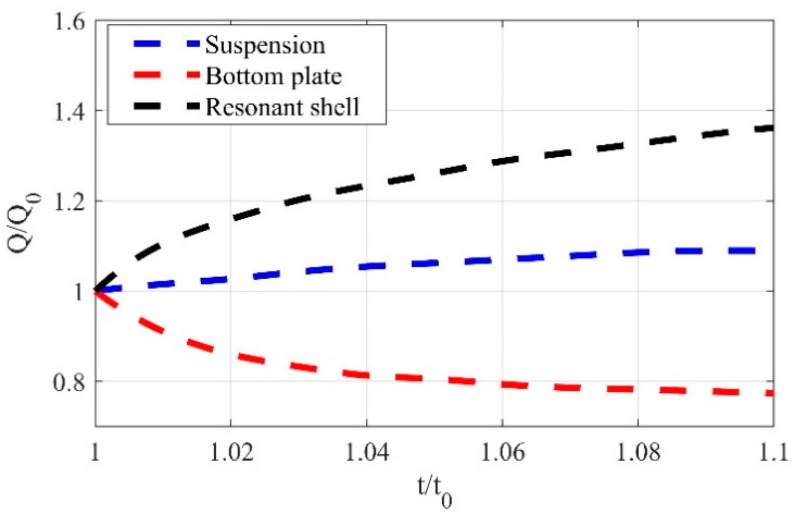
Effect of structural thickness on Q_TED_.

Based on the above hypothesis, the inner diameters of the resonant shell and the suspension are fixed at their initial values, while the thicknesses of the resonant shell, the suspension and the bottom plate change by 10% of their original size, respectively. The Q_TED_ of the initial structure is defined as Q_0_, and the thickness of the resonant shell, the suspension and the bottom plate is defined as t_0_. Figure 5 shows the normalized Q_TED_, defined as Q/Q_0_, versus the change in the normalized thickness of the resonant shell, the suspension and the bottom plate, defined as t/t_0_.

As depicted in Figure 5, the thermal resistance in the radial hot and cold region increases with the increase in thickness of the resonant shell and the suspension, resulting in improved Q_TED_. In addition, thermal resistance in the axial hot and cold region increases with the thickness of the bottom plate, but the area of heat conduction on the azimuth also increases; that is, the thermal resistance decreases. According to the variation trend of the Q/Q_0_ with the thickness of the bottom plate, it can be observed that the irreversible heat flow in the azimuthal direction of the bottom plate plays a more important role than the irreversible heat flow across the thickness of the bottom plate on Q_TED_. Additionally, compared with the resonant shell and the bottom plate, the suspension has less influence on the TED. Therefore, in order to improve the Q_TED_ of the cylindrical resonator, the structure of the resonant shell and bottom plate should be mainly adjusted. Considering the variation trend of Q/Q_0_ with the thickness of the resonant shell and considering other losses such as anchor loss, the thickness of the resonant shell and the bottom plate are finally designed to be 1.0 mm and 0.4 mm, respectively. The eigenfrequency and the Q_TED_ of the improved structure are found to be 6741.6 Hz and 1.41 × 10^9^, respectively, and the Q_TED_ of the improved structure is 73% higher than that of the initial structure.

### 2.3. Effect of The Bottom Plate Shape

**Figure 6:**
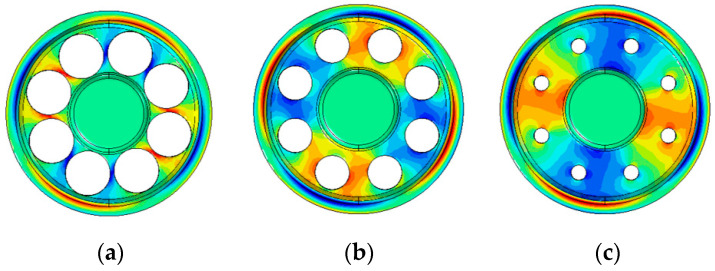
Temperature gradient distribution of the cylindrical resonator with different bottom holes. (**a**) The resonator with 6 mm bottom holes; (**b**) The resonator with 4 mm bottom holes; (**c**) The resonator with 2 mm bottom holes.

It is known that adding holes on a vibrating structure may reduce the thermal elastic damping [38,39]. Considering the smaller deformation of the bottom plate and the consistency of the manufacturing process, the bottom holes are designed to achieve the goal of improving the Q_TED_. The temperature gradient distribution of a resonator with eight bottom holes of different diameters is shown in Figure 6. It can be observed in the figure that the holes in the bottom plate increase the thermal resistance on the bottom plate during vibration, which reduces the irreversible heat transfer, thereby increasing Q_TED_.

**Figure 7:**
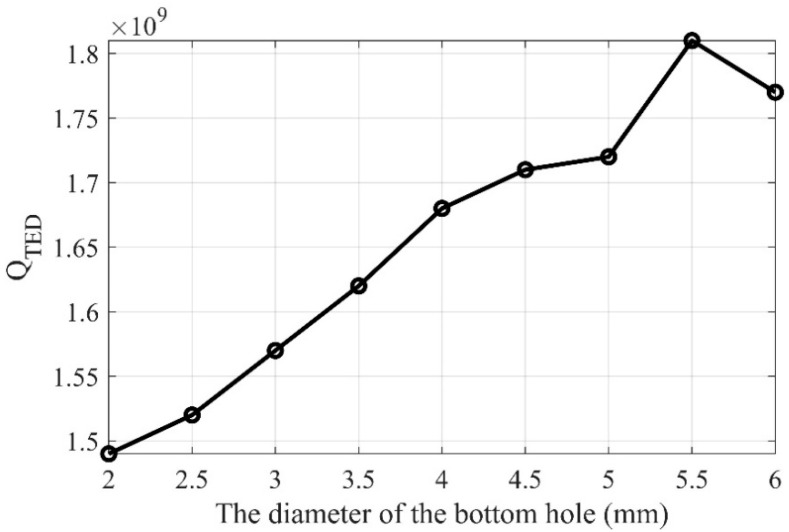
The Q_TED_ varies with the diameter of the bottom hole.

The Q_TED_ of cylindrical resonators with different diameters of bottom holes are simulated, and the results are depicted in Figure 7. Within a range of 2 to 5.5 mm, larger bottom holes yield a significant increase in Q_TED_. Therefore, the bottom hole is designed with a diameter of 5.5 mm to reduce the TED of the cylindrical resonator, and the eigenfrequency and Q_TED_ of the improved structure are found to be 6571.9 Hz and 1.81 × 10^9^, respectively. The Q_TED_ of the improved structure is 122% higher than the Q_TED_ of the initial structure when the bottom hole diameter is merely 3 mm.

## 3. Experimental Result

**Figure 8:**
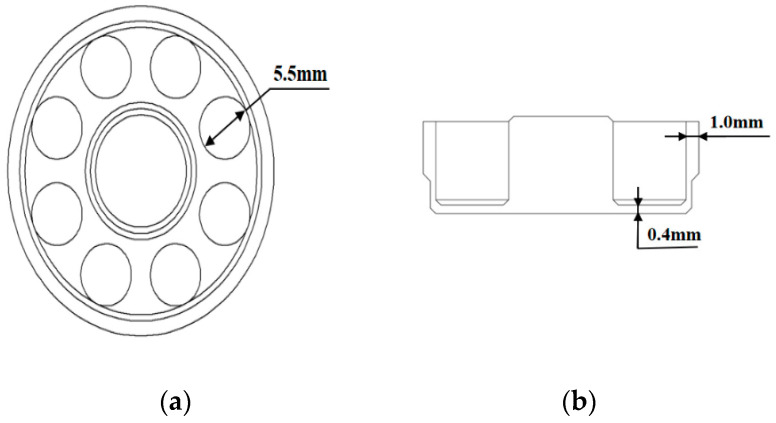
Schematic of: (**a**) The top view of the improved cylindrical resonator; (**b**) The sectional view of the improved cylindrical resonator.

The simulation results indicate that a proper adjustment of the resonator shell structure can effectively improve the Q_TED_. Besides, the existence of bottom holes can also improve the Q_TED_ significantly. Therefore, the thickness of the resonant shell, the bottom plate and the diameter of the bottom holes are designed to be 1.0, 0.4 and 5.5 mm, respectively, as shown in Figure 8. Cylindrical resonators with improved structures fabricated with a similar process have been reported before [40], and post-fabrication treatments, including annealing and chemical etching [23,24], have been applied to these resonators.

**Figure 9:**
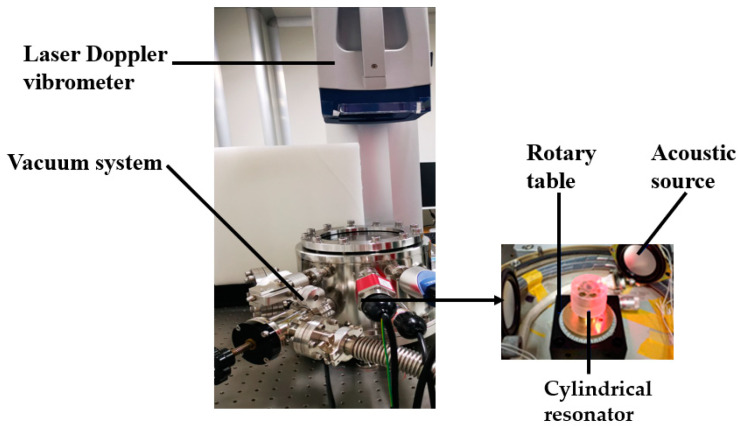
The vibration characteristic measuring system.

The Q factor of the improved resonator is experimentally tested. The process of measurement has been described in detail in our previous study [23]. As shown in Figure 9, the experimental devices includes a laser Doppler vibrometer and a vacuum system. When the Q factor of the resonator is measured at 1 atm, the Q factor is low duo to the influence of air damping. Therefore, the Q factor can be calculated by Equation (5) [23]:

(5) $$Q=\frac{f}{\Delta f}$$

where *f* represents the resonant frequency and Δf represents the −3 dB bandwidth of the frequency response of the resonator. For a much higher Q factor, the ring-down time method is employed to calculate the Q factor:

(6) Q=πfτ

where *f* represents the resonant frequency and τ represents the ring-down time when the amplitude attenuates to 1/e of the initial value.

**Figure 10:**
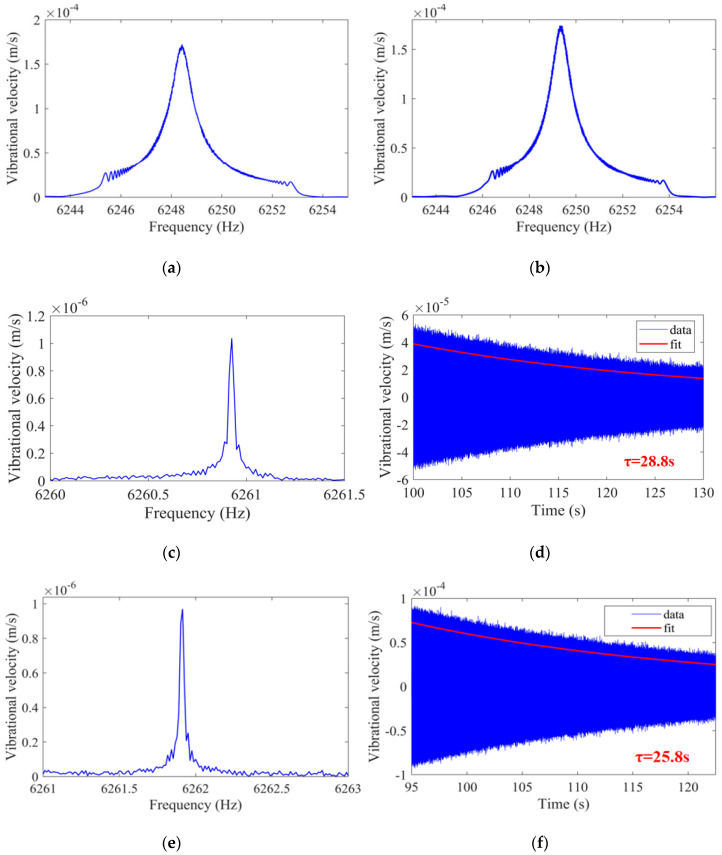
Schematic of: (**a**) The frequency response of the low-frequency axis after annealing is measured at 1 atm; (**b**) The frequency response of the high-frequency axis after annealing is measured at 1 atm; (**c**) The frequency response of the low-frequency axis after annealing is measured at 0.01 Pa; (**d**) The decay time of the low-frequency axis after annealing is measured at 0.01 Pa; (**e**) The frequency response of the high-frequency axis after annealing is measured at 0.01 Pa; (**f**) The decay time of the high-frequency axis after annealing is measured at 0.01 Pa.

A proper annealing process improves the structural temperature of the cylindrical resonator. The residual internal stress of the material and the pollutants on the cylindrical resonator will be removed by the annealing process, thus improving the Q factor [41,42]. The specific process of annealing is presented in Table 2 [40]. After annealing, the resonant frequency and Q factor of the improved cylindrical resonator are measured at 1 atm and 0.01 Pa, respectively. The frequency responses of the resonator at 1 atm are depicted in Figure 10a,b. The resonant frequencies of the resonator are 6248.4 and 6249.3 Hz, and Q factors are 7980 and 6249, respectively. When the Q factor is measured at 0.01 Pa, Equation (6) is used for the Q calculation. The vibration data measured by laser Doppler vibrometer are fitted exponentially, and the results of the resonator after annealing are depicted in Figure 10. The results indicate that the resonant frequency of the low-frequency axis is 6260.9 Hz, and the decay time is 28.8 s, which gives a Q factor of 5.66 × 10^5^. The resonant frequency of the high-frequency axis is 6261.9 Hz, and the decay time is 25.8 s, which gives a Q factor of 5.07 × 10^5^.

**Figure 11:**
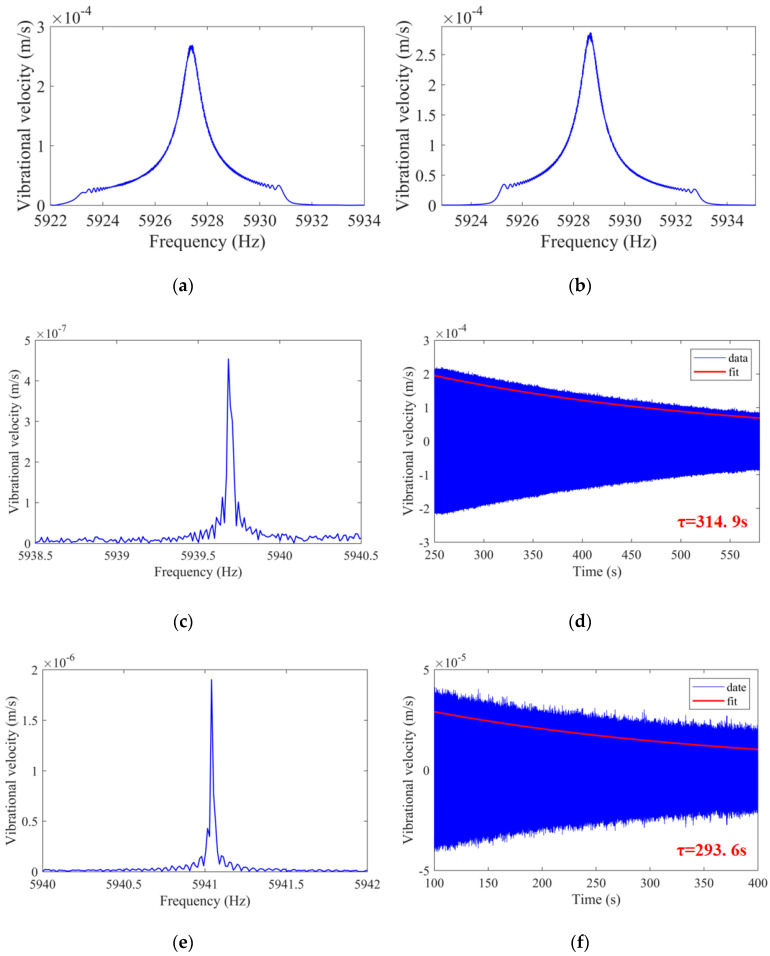
Schematic of: (**a**) The frequency response of the low-frequency axis after chemical etching is measured at 1 atm; (**b**) The frequency response of the high-frequency axis after chemical etching is measured at 1 atm; (**c**) The frequency response of the low-frequency axis after chemical etching is measured at 0.01 Pa; (**d**) The decay time of the low-frequency axis after chemical etching is measured at 0.01 Pa; (**e**) The frequency response of the high-frequency axis after chemical etching is measured at 0.01 Pa; (**f**) The decay time of the high-frequency axis after chemical etching is measured at 0.01 Pa.

After annealing, surface defects and surface impurities caused by mechanical processing still exist on the resonator. These imperfects will cause the Q factor to be severely limited [43,44,45,46,47,48,49]. In order to reduce the surface loss of the resonator, NH_4_F_2_ is used as the etching solution to chemically etch the cylindrical resonator [50]. After chemical etching, the resonant frequency and Q factor of the improved cylindrical resonator are measured at 1 atm and 0.01 Pa, respectively. The frequency responses of the resonator at 1 atm are depicted in Figure 11a,b. The resonant frequencies of the resonator are 5927.4 and 5928.6 Hz, which give Q factors of 8461 and 8428, respectively. The results of the chemically etched resonator at 0.01 Pa are depicted in Figure 11. The resonant frequency of the low-frequency axis decreases to 5939.7 Hz while the decay time increases to 314.9 s, which gives a Q factor of 5.86 × 10^6^. The resonant frequency of the high-frequency axis is 5941.0 Hz, and the decay time is 293.6 s, which gives a Q factor of 5.48 × 10^6^. To our knowledge, this is the highest Q factor reported in cylindrical resonators to date.

## 4. Discussion

The simulation results revealed that the geometric structure of the cylindrical resonator had a significant influence on the Q_TED_. The irreversible heat flow transfer of the cylindrical resonator in the n = 2 mode was mainly influenced by the structure and thickness of the bottom plate and resonant shell. The variation of Q_TED_ of the cylindrical resonator was stimulated by changing the structure of the bottom plate and the resonant shell, respectively. After that, the thickness of the resonant shell and the bottom plate was adjusted, and 5.5 mm-diameter bottom holes were designed to improve the Q_TED_. The improved cylindrical resonator was then manufactured and processed with the same annealing and chemical etching process, as previously reported [23,24]. The resonant frequency and the Q factor of the improved cylindrical resonator after annealing and chemical etching in the medium vacuum were then measured, respectively. The experimental results illustrated that the Q factor of the cylindrical resonator was significantly increased, achieving 5.86 million. The reason for the significant increase in the Q factor may be as follows: the thermoelastic dissipation of the cylindrical resonator was significantly reduced, the anchor loss was suppressed and the bottom holes reduced the surface loss by affecting the surface-to-volume ratio of the resonator. Note that the frequency obtained by simulation was slightly different from that measured in the experiment and that the Q_TED_ of the cylindrical resonator obtained in the simulation was three orders of magnitude higher than the Q factor obtained in the experiment. This might be due to the simplification of the simulation modal in the aspects of clamping condition, internal friction, surface defects and surface roughness of the resonator. From the above experimental results, it can be obtained that the Q factor measured at 1 atm differed by three orders magnitude from the Q factor measured at 0.01 Pa. The effect of air damping was not considered in the simulation results. Therefore, air damping is also a significant factor that causes a difference between simulation and experimental results. Through the comparison of simulation results and experimental results, it should be pointed out that this simulation modal is more suitable for revealing the variation trend of Q_TED_ with different cylindrical resonator structures.

## 5. Conclusions

In this paper, by simulating the thermoelastic dissipation of the cylindrical resonator, the main directions of irreversible heat flow were obtained in the vibration of the cylindrical resonator structure. Furthermore, the simulation results illustrated that compared with the suspension, the resonant shell and the bottom plate had a more significant influence on the TED. By analyzing the results of the simulation, the effects of different resonant shell and bottom plate structures were investigated. Through a series of simulations of the geometric structure of the resonator, the improved structure with Q_TED_ 122% higher than that of the initial structure was finally obtained. During the experiment, the resonant frequency and Q factor of the cylindrical resonator were measured after annealing and chemical etching, respectively. The Q factor of the improved cylindrical resonator achieved 5.86 million, the highest value of the Q factor reported in cylindrical resonators to date. Moreover, for the cylindrical resonator structure proposed in this paper, the manufacturing process, annealing treatment and chemical etching could be optimized to achieve even higher Q factors in the future. Cylindrical resonators are not only expected to be used in low and medium precision inertial navigation systems but should also have the potential to be used in high-precision inertial navigation systems at a lower cost.

## Figures and Tables

**Table 1:** Physical properties of fused silica.

Component	Value	Units
Young’s modulus (E)	72	GPa
Density (ρ)	2200	kg/m^3^
Specific heat capacity (C)	770	J/(kg K)
Thermal conductivity (k)	1.38	W/(m K)
Coefficient of thermal expansion (α)	5.2 × 10^−7^	1/K
Poisson’s ratio (ν)	0.18	

**Table 2:** The process of annealing.

Annealing Process	Heating Process		Heat Preservation	Cooling Process	
	Temperature	Heating Rate	Temperature/Time	Temperature	Cooling Rate
Parameter	<800 °C	10 °C/min	1150 °C	900 °C	0.5 °C/min
	1150 °C	4 °C/min	8 h	≤800 °C	Free cooling

## References

[1] Chikovani V., Okon I.M., Barabashov A., Tewksbury P. A set of high accuracy low cost metallic resonator CVG. Proceedings of the 2008 IEEE/ION Position, Location and Navigation Symposium.

[2] Chikovani V., Yatsenko Y., Barabashov A., Marusyk P., Umakhanov E., Taturin V. (2009). Improved accuracy metallic resonator CVG. IEEE Aerosp. Electron. Syst. Mag..

[3] Chikovani V.V., Yatzenko Y.A., Kovalenko V.A. (2009). Coriolis Force Gyroscope with High Sensitivity.

[4] Rozelle D.M., Segerman A.M., Lai P.C., Wilkins M.P., Pittelkau M.E. (2009). The Hemispherical Resonator Gyro: From Wineglass to the Planets. Spaceflight Mechanics, PTS I-III.

[5] Sarapuloff S. Development and cost reduction of high-Q dielectric resonators of solid-state gyroscopes. Proceedings of the 8th Saint Petersburg International Conference on Integrated Navigation Systems.

[6] Sarapuloff S.A. In Dynamics of Precise Solid-State Gyroscopes of HRG and CRG Types. Proceedings of the V-th Brazilian Symposium on Inertial Engineering (V SBEIN).

[7] Watson W.S. Vibratory gyro skewed pick-off and driver geometry. Proceedings of the IEEE/ION Position, Location and Navigation Symposium.

[8] Yatsenko Y.A., Petrenko S., Vovk V., Chikovani V. Technological aspects of manufacturing of compound hemispherical resonators for small-sized vibratory gyroscopes. Proceedings of the 6 th Saint Petersburg International Conference on Integrated Navigation Systems.

[9] Northrop Grumman Corporation. https://news.northropgrumman.com/news/releases/northrop-grummans-hemispherical-resonator-gyro-achieves-50-million-operating-hours-in-space.

[10] Jeanroy A., Leger P. (2002). Gyroscopic Sensor and Apparatus for Measuring Rotation Using Same.

[11] Delhaye F. In *HRG by SAFRAN:* The game-changing technology. Proceedings of the 2018 IEEE International Symposium on Inertial Sensors and Systems (INERTIAL).

[12] Foloppe Y., Lenoir Y. HRG Crystal™ DUAL CORE: Rebooting the INS revolution. Proceedings of the 2019 DGON Inertial Sensors and Systems (ISS).

[13] Singh S., Nagourney T., Cho J.Y., Darvishian A., Shiari B., Najafi K. Design and fabrication of high-Q birdbath resonator for mems gyroscopes. Proceedings of the 2018 IEEE/ION Position, Location and Navigation Symposium (PLANS).

[14] Asadian M.H., Wang D., Wang Y., Shkel A.M. 3D dual-shell micro-resonators for harsh environments. Proceedings of the 2020 IEEE/ION Position, Location and Navigation Symposium (PLANS).

[15] Cho J.Y., Singh S., Woo J., He G., Najafi K. 0.00016 deg/√hr Angle Random Walk (ARW) and 0.0014 deg/hr Bias Instability (BI) from a 5.2M-Q and 1-cm Precision Shell Integrating (PSI) Gyroscope. Proceedings of the 2020 IEEE International Symposium on Inertial Sensors and Systems (INERTIAL).

[16] Zhuravlev V.P. (2015). Hemispherical resonator gyro with m data electrodes and n control electrodes. Mech. Solids.

[17] Wei Z., Yi G., Huo Y., Qi Z., Xu Z. (2019). The Synthesis Model of Flat-Electrode Hemispherical Resonator Gyro. Sensors.

[18] Pi J., Bang H. (2013). Imperfection Parameter Observer and Drift Compensation Controller Design of Hemispherical Resonator Gyros. Int. J. Aeronaut. Space Sci..

[19] Trusov A.A., Meyer A.D., Mccammon G.H., Bettadapura A., Philips M.R. Toward software defined coriolis vibratory gyroscopes with dynamic self-calibration. Proceedings of the 2016 DGON Intertial Sensors and Systems (ISS).

[20] Xu Z., Xi B., Yi G., Wang D. (2020). A Novel Model for Fully Closed-loop System of Hemispherical Resonator Gyroscope under Force-to-Rebalance Mode. IEEE Trans. Instrum. Meas..

[21] Xu Z., Yi G., Er M.J., Huang C. (2019). Effect of Uneven Electrostatic Forces on the Dynamic Characteristics of Capacitive Hemispherical Resonator Gyroscopes. Sensors.

[22] Luo Y., Gebrel I., Bognash M., Pan Y., Liu F., Asokanthan S., Luo H., Qu T. (2020). Dynamic Response and Frequency Split Predictions for Cylindrical Fused Silica Resonators. IEEE Sens. J..

[23] Pan Y., Wang D., Wang Y., Liu J., Wu S., Qu T., Yang K., Luo H. (2016). Monolithic Cylindrical Fused Silica Resonators with High Q Factors. Sensors.

[24] Luo Y., Qu T., Cui Y., Pan Y., Yu M., Luo H., Jia Y., Tan Z., Liu J., Zhang B. (2019). Cylindrical Fused Silica Resonators Driven by PZT Thin Film Electrodes with Q Factor Achieving 2.89 Million after Coating. Sci. Rep..

[25] Moeenfard H., Ahmadian M.T., Farshidianfar A. (2012). Modeling squeezed film air damping in torsional micromirrors using extended Kantorovich method. Meccanica.

[26] Yasumura K., Stowe T., Chow E., Pfafman T., Kenny T., Stipe B., Rugar D. (2000). Quality factors in micron- and submicron-thick cantilevers. J. Microelectromech. Syst..

[27] Hao Z., Erbil A., Ayazi F. (2003). An analytical model for support loss in micromachined beam resonators with in-plane flexural vibrations. Sens. Actuators A Phys..

[28] Darvishian A., Shiari B., He G., Najafi K. Effect of substrate thickness on quality factor of mechanical resonators. Proceedings of the2015 IEEE International Symposium on Inertial Sensors and Systems (ISISS).

[29] Ghaffari S., Kenny T.W. (2012). Thermoelastic Dissipation in Composite Silicon MEMS Resonators with Thin Film Silicon Dioxide Coating Mrs Online. Proc. Libr..

[30] Zener C. (1938). Internal Friction in Solids II. General Theory of Thermoelastic Internal Friction. Phys. Rev..

[31] Braginsky V.B., Mitrofanov V.P., Panov V.I., Hetherington J.H. (1987). Systems with Small Dissipation (First published in Moscow in 1981 as “Sistemis Maloi Dissipatsiei”) by V. B. Braginsky, V.P. Mitrofanov, and V. I. Panov (Translated by Erast Gliner). J. Acoust. Soc. Am..

[32] Shiari B., Nagourney T., Darvishian A., Cho J.Y., Najafi K. Numerical study of impact of surface roughness on thermoelastic loss of micro-resonators. 2017 IEEE International Symposium on Inertial Sensors and Systems (INERTIAL).

[33] Sorenson L., Shao P., Ayazi F. (2014). Bulk and Surface Thermoelastic Dissipation in Micro-Hemispherical Shell Resonators. J. Microelectromech. Syst..

[34] Darvishian A., Nagourney T., Cho J.Y., Shiari B., Najafi K. (2017). Thermoelastic Dissipation in Micromachined Birdbath Shell Resonators. J. Microelectromech. Syst..

[35] Hao Z., Ayazi F. (2005). Thermoelastic Damping in Flexural-Mode Ring Gyroscopes. Adv. Bioeng..

[36] Hamza A., Tsukamoto T., Tanaka S. Quality factor trimming method using thermoelastic dissipation for ring-shape MEMS resonator. Proceedings of the 2020 IEEE International Symposium on Inertial Sensors and Systems (INERTIAL).

[37] Moeenfard H., Kaji F., Ahmadian M.T. Coupled bending and torsion effects on the squeezed film air damping in torsional micromirrors. Proceedings of the 6th International Conference on Micro- and Nanosystems; 17th Design for Manufacturing and the Life Cycle Conference.

[38] Bernstein J.J., Bancu M.G., Bauer J.M., Cook E.H., Kumar P., Newton E., Nyinjee T., Perlin G.E., Ricker J.A., Teynor W.A. (2015). High Q diamond hemispherical resonators: Fabrication and energy loss mechanisms. J. Micromech. Microeng..

[39] Vafanejad A., Kim E.S. Effect of diaphragm perforation on quality factor of hemispherical resonator gyroscope. Proceedings of 2015 Transducers–2015 18th International Conference on Solid-State Sensors, Actuators and Microsystems (TRANSDUCERS).

[40] Luo Y., Pan Y., Zhou G., Qu T., Luo H., Zhang B. Annealing experiments on the quality factor of fused silica cylindrical shell resonator. Proceedings of the 2019 IEEE International Symposium on Inertial Sensors and Systems (INERTIAL).

[41] Ahamed M.J., Senkal D., Shkel A.M. Effect of annealing on mechanical quality factor of fused quartz hemispherical resonator. Proceedings of the 2014 International Symposium on Inertial Sensors and Systems (ISISS).

[42] Nagourney T., Cho J.Y., Darvishian A., Shiari B., Najafi K. Effect of metal annealing on the Q-factor of metal- coated fused silica micro shell resonators. Proceedings of the 2015 IEEE International Symposium on Inertial Sensors and Systems (ISISS) Proceedings; Institute of Electrical and Electronics Engineers (IEEE).

[43] Li S., Wang Z., Wu Y. (2008). Relationship between subsurface damage and surface roughness of optical materials in grinding and lapping processes. J. Mater. Process. Technol..

[44] Davis K., Tomozawa M. (1995). Water diffusion into silica glass: Structural changes in silica glass and their effect on water solubility and diffusivity. J. Non-Cryst. Solids.

[45] Hed P.P., Edwards D.F. (1987). Optical glass fabrication technology 2: Relationship between surface roughness and subsurface damage. Appl. Opt..

[46] Miller P.E., Suratwala T., Wong L.L., Feit M.D., Menapace J.A., Davis P.J., Steele R.A. The distribution of subsurface damage in fused silica. Proceedings of the Boulder Damage Symposium XXXVII: Annual Symposium on Optical Materials for High Power Lasers.

[47] Mitrofanov V., Tokmakov K. (2003). Effect of heating on dissipation of mechanical energy in fused silica fibers. Phys. Lett. A.

[48] Suratwala T., Wong L., Miller P., Feit M., Menapace J., Steele R., Davis P., Walmer D. (2006). Sub-surface mechanical damage distributions during grinding of fused silica. J. Non-Cryst. Solids.

[49] Suratwala T., Steele R., Feit M., Wong L., Miller P., Menapace J., Davis P. (2008). Effect of rogue particles on the sub-surface damage of fused silica during grinding/polishing. J. Non-Cryst. Solids.

[50] Zhai Y.J., Pan Y., Jia Y.L., Liu J.P., Tan Z.Q., Yang K.Y., Luo H. Surface evolution of cylindrical fused silica resonator and its implication on Q factor. Proceedings of the 2019 DGON Inertial Sensors and Systems (ISS).

